# Fast Degradation of Bisphenol A in Water by Nanostructured CuNPs@CALB Biohybrid Catalysts

**DOI:** 10.3390/nano10010007

**Published:** 2019-12-18

**Authors:** Noelia Losada-Garcia, Alba Rodriguez-Otero, Jose M. Palomo

**Affiliations:** Department of Biocatalysis, Institute of Catalysis (CSIC), Marie Curie 2, Cantoblanco, Campus UAM, 28049 Madrid, Spain; n.losada@csic.es (N.L.-G.); alba.rodriguez.96@gmail.com (A.R.-O.)

**Keywords:** Bisphenol A, nanostructure, copper, nanobiohybrids, degradation

## Abstract

Copper nanoparticles–enzyme biohybrid is a promising material for the remediation of contaminated waters, but its function is influenced by its effect on degradation organic pollutants. This study is the first investigation into the fast degradation of a high amount of Bisphenol A (BPA) in water at neutral pH and room temperature. Four different CuNPs biohybrids with different cu species and nanoparticle sizes were used as catalysts. The biohybrid **CuNPs@CALB-3**, which contained Cu_2_O nanoparticles of around 10 nm size, showed excellent catalytic performance removing >95% BPA content (45 ppm) in an aqueous solution in 20 min in the presence of hydrogen peroxide at pH 8 using 1.5 g/L of a catalyst. The catalyst showed excellent stability and recyclability at these conditions.

## 1. Introduction

In recent decades, natural water resources have been contaminated with organic and inorganic pollutants, such as polyaromatic hydrocarbons, dyes or heavy metals [1]. The risk to the health of the human body of these pollutants is well known, in particular their presence in drinking water above a certain limit. This is the motive why the elimination of these pollutants from water is a major research topic at present [2].

In particular, Bisphenol A (BPA) is a very significant building block in the fabrication of food cans, polycarbonate plastics and other daily used chemicals [3,4,5,6]. However, the regular and global usage of BPA and BPA-contained products has given rise to its omnipresent delivery in water, sediments or atmosphere. This molecule has been recognized as an environmental endocrine disruptor for its estrogenic and genotoxic activity [7]. Consequently, BPA contamination in the environment is a progressively worldwide alarm, and methods to ably remove it from the environment are urgently recommended. In 2018, the European Commission adopted a proposal to strengthen the regulation on the use of BPA in food contact materials, the new regulation introduces a new specific migration limit (SML) of 0.05 ppm of BPA [8].

Therefore, a methodology to obtain a very fast, efficient and green strategy to eliminate BPA is mandatory.

Different strategies have been described in literature; the most recent successful application has been the use of metal nanoparticles as a catalyst [9,10,11,12,13,14,15,16].

The high surface-to-volume ratio of nanomaterials compared to bulk materials is one of the main advantages of nanoparticles as catalysts [17].

In particular, most examples have been developed using iron nanomaterials as catalysts [9,10,11,12,13] and only few examples using Cu or Cu-based materials have been applied as catalysts in this reaction [14,15,16,17,18,19,20]. For example, Zhao and coworkers^14^ described the degradation of 87% BPA (20 ppm) in 180 min using a Cu–Al_2_O_3_ membrane in the presence of hydrogen peroxide at neutral pHs.

Therefore, from an applicable point of view, the preparation of heterogeneous highly-active, selective, stable, and robust Cu nanocatalysts is required. The reuse and stability of the catalyst are quite important in the application of this system to remediation [18,19,20].

**Figure 1:**
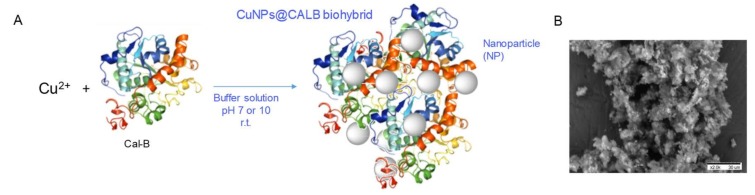
(**A**) Schematic illustration of the fabrication of CuNPs@CALB biohybrids. (**B**) SEM image of CuNPs@CALB biohybrids.

Herein, we have developed a very efficient and green methodology to completely remove BPA (45 ppm) from water solution at room temperature based on the application of a set of different nanostructured copper materials as catalysts. These heterogeneous nanostructured materials—synthesized by a very green and efficient method—are formed by Cu nanoparticles (different copper species depending synthetic protocol) embedded on a protein matrix as a heterogeneous form (Figure 1) that is quite stable, being reused several times without losing the catalytic efficiency, which is a critical role for industrial application.

## 2. Materials and Methods

### 2.1. Materials

Lipase B from *Candida antarctica* (CAL-B) solution was purchased from Novozymes (Copenhagen, Denmark). Copper (II) sulfate pentahydrate [Cu_2_SO_4_ 5H_2_O] and hydrogen peroxide (33%) were from Panreac (Barcelona, Spain). Sodium bicarbonate, sodium phosphate, sodium borohydride, Bisphenol A (BPA), benzoquinone, and hydroquinone were purchased from Sigma-Aldrich (St. Louis, MO, USA). HPLC grade acetonitrile was purchased from Scharlab (Barcelona, Spain).

### 2.2. Fabrication of CuNPs@CAL-B Nanobiohybrids

1.8 mL of commercial (18 mg of protein) *Candida antarctica* lipase solution was added to 60 mL sodium phosphate (100 mM, pH 7) in a 250 mL glass bottle containing a small magnetic bar stirrer. Then, 600 mg of Cu_2_SO_4_∙5H_2_O (10 mg/mL) was added to the protein solution and it was maintained for 16 h. After the first 30 min incubation, the solution turned cloudy (turquoise) and the pH solution was measured indicating a decrease to pH 6. After 16 h incubation, the mixture was centrifuged at 8000 rpm for 5 min using a Biocen 22 R (Orto-Alresa, Ajalvir, Spain) refrigerated centrifuge. The generated pellet was re-suspended in 15 mL of water and centrifuged. This step was repeated three times more. Then, the solid was re-suspended in 2 mL of water added to a cryogenic tube frozen with liquid nitrogen (approx. 5 min) and lyophilized for 16 h. After that, 150 mg of the so called **CuNPs@CALB-1** was obtained.

The same protocol was used but after 16 h incubation, 6 mL of NaBH_4_ (300 mg) aqueous solution (1.2 M) was added to the cloudy solution (in two times of 3 mL) obtaining a final concentration of 0.12 M of sodium borohydride in the mixture. The solution turned rapidly black, and the mixture was reduced during 40 min. After that, the solid was washed and frozen as previously described. After the lyophilization process, around 150 mg of the so called **CuNPs@CALB-2** was obtained. The protocol in this second catalyst (including reduction step) was repeated using double the amount of enzyme (3.6 mL instead of 1.8 mL). In this case, after the lyophilization step, the so called **CuNPs@CALB-3** was obtained. Finally, another modification in the synthetic protocol consisted of the modification of the initial pH, using sodium bicarbonate (100 mM, pH 10) as buffer solution. In this case, after lyophilization, more than 100 mg of the so called **CuNPs@CALB-4** was obtained.

### 2.3. Characterization Techniques

Inductively coupled plasma-optical emission spectrometry (ICP-OES) was performed on an OPTIMA 2100 DV instrument (PerkinElmer, Waltham, MA, USA). X-Ray diffraction (XRD) patterns were obtained using a Texture Analysis D8 Advance Diffractometer (Bruker, Billerica, MA, USA) with Cu Kα radiation. The TEM and HRTEM images were obtained on a 2100F microscope (JEOL, Tokyo, Japan) equipped with an EDX detector INCA x-sight (Oxford Instruments, Abingdon, UK).

### 2.4. Water Treatment: Active CuNPs@CAL-B Nanobiohybrids Catalyzing the Degradation of Bisphenol A (BPA)

A solution of 10 mM of BPA in pure acetonitrile was prepared; 0.2 mL of this solution were dissolved in 10 mL of either 100 mM or 5 mM sodium phosphate buffer pH 6, pH 7 or pH 8 to achieve a 0.2 mM concentration of BPA (45 mg L^−1^). The solution pH was adjusted using HCl or NaOH 1 M. Then, hydrogen peroxide (33% *v/v*) was added to this BPA solution to obtain different concentrations (12, 25, 50, 100, or 150 mM). To initialize the reaction, 3 mg of the catalyst was added to 2 mL of this solution (BPA and H_2_O_2_) in a 7 mL glass flask. Gentle stirring was provided at room temperature by a roller. At given time intervals, 30 µL of the reaction solution was taken and diluted five times in acetonitrile/water (50/50 *v/v*) mixture for HPLC analysis. For each degradation group, three parallel experiments were performed, and the error range was <5%. The HPLC analysis was performed on spectrum P100 HPLC system (Thermo Scientifics, Waltham, MA, USA) HPLC equipment, which was equipped with a C8 Kromasil reversed phase column (150 mm × 4.6 mm, 5 μm, Analisis Vinicos) and a UV6000LP detector (λ = 225 nm). The mobile phase was an acetonitrile/water (50/50 *v/v*) mixture, and the flow rate was 1 mL min^−1^. Under these conditions, the retention time of BPA was 4.90 min, and for H_2_O_2_ was 1.57 min. Products from BPA degradation such as benzoquinone or hydroquinone were detected at 2.7 min and 2.1 min, using pure standard substrates as a control.

TOF (turnover frequency) value was calculated by mmols BPA converted/mmols Cu^−1^ × time (h^−1^).

### 2.5. Reuse of CuNPs@CALB-3 Nanobiohybrids in the Degradation of Bisphenol A (BPA)

The **CuNPs@CALB-3** biohybrid was reused in five cycles for the degradation of BPA using the same experimental conditions described above. After each reaction cycle, the catalyst was washed with water several times, centrifuged and separated before the next reaction.

### 2.6. Catalase-Like Activity of Cu Nanobiohybrids

A substrate solution was prepared adding 52 µL of hydrogen peroxide to 9.8 mL of 100 mM or 5 mM phosphate buffer (pH 6, pH 7 and pH 8) or distilled water in order to obtain a final concentration of 50 mM. The solution pH was adjusted using HCl or NaOH 1 M. To start the reaction, 4.5 mg of the Cu nanobiohybrid was added to 3 mL of the previous solution at room temperature. The reaction was followed by measuring the degradation of hydrogen peroxide recording the decrease of absorbance spectrophotometrically at 240 nm in quartz cuvettes of 1-cm path length, adding 2 mL of this solution at different times. After each measurement, the volume added was again recovered and poured in to the reaction solution.

## 3. Results

### 3.1. Synthesis and Caractherization of Nanostructured CuNPs@CAL-B Nanobiohybrids Catalysts

**Scheme 1:**
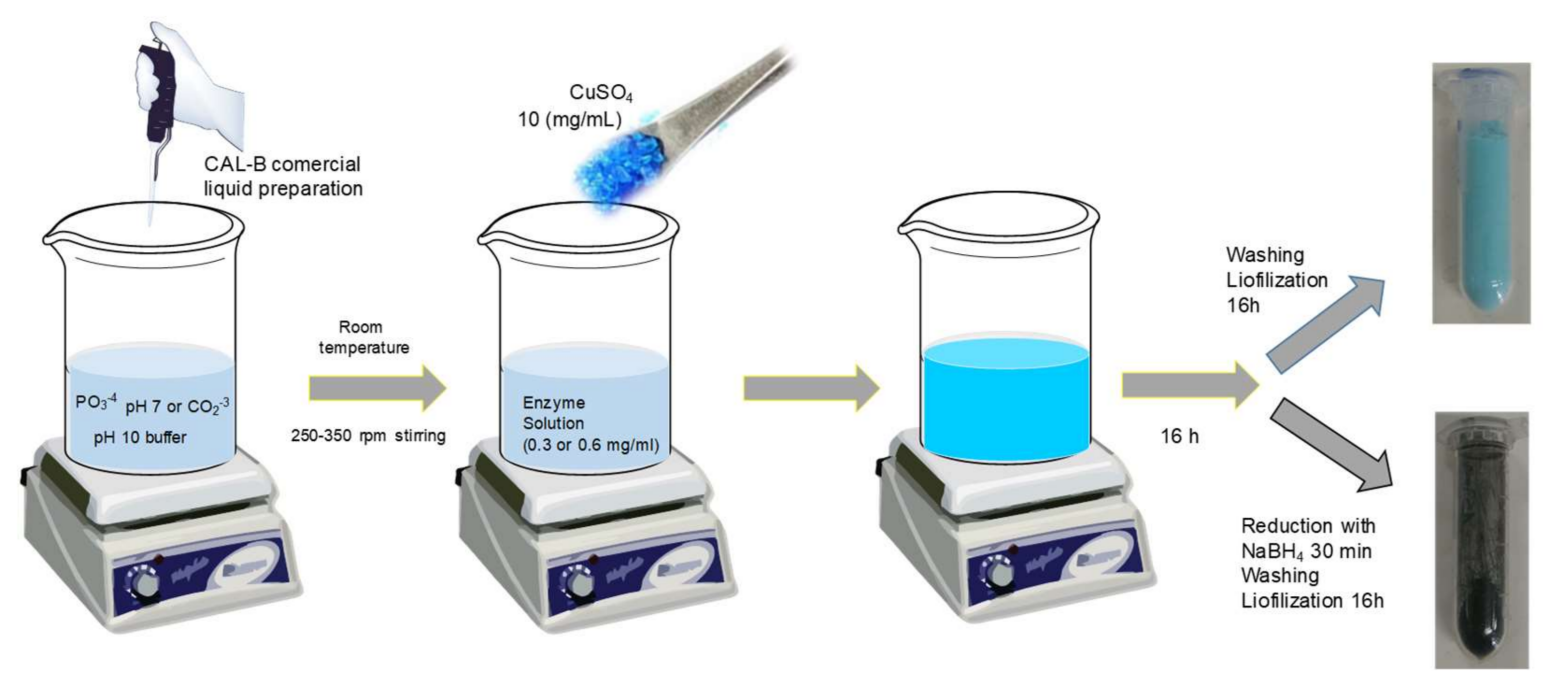
Protocol of synthesis of CuNPs@CAL-B nanobiohybrids.

The fabrication of nanostructured CuNPs@CAL-B biohybrid is shown in Figure 1 and Scheme 1. A liquid commercial solution of lipase B from *Candida antarctica* (CALB, 33 kDa) [21] dissolved in phosphate pH 7 or bicarbonate pH 10 was mixed with copper sulfate at room temperature and incubated under magnetic stirring during 16 h. After that, the formed solid was chemically reduced or not, washed and lyophilized following the steps described in the experimental section.

**Figure 2:**
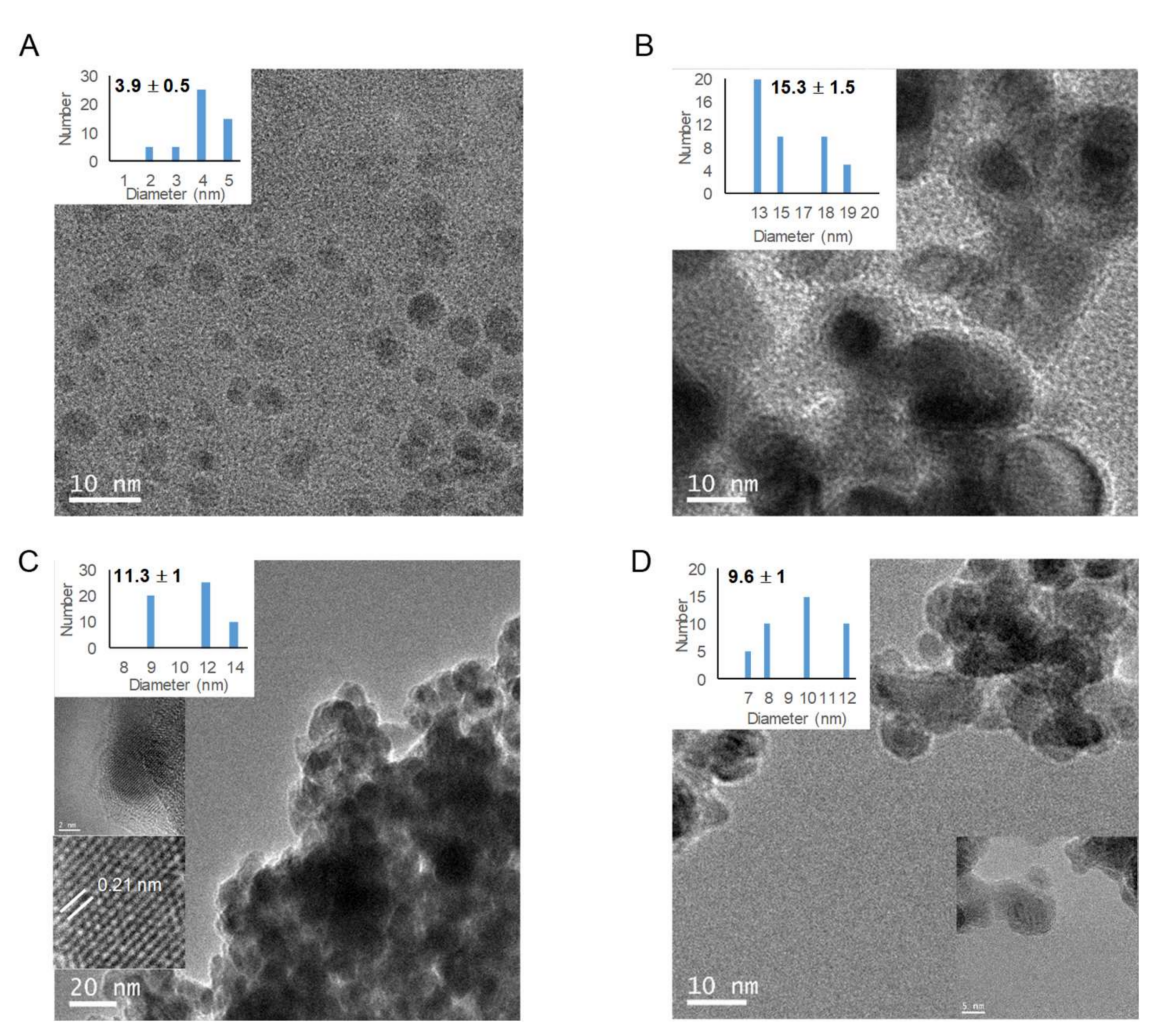
TEM images of the different CuNPs@CALB biohybrids. (**A**) **CuNPs@CALB-1**, (**B**) **CuNPs@CALB-2**, (**C**) **CuNPs@CALB-3** (HRTEM inset), (**D**) **CuNPs@CALB-4**. Particle size distribution profile (inset).

The TEM analysis demonstrated the formation of homogeneously distributed nanoparticles in all cases (Figure 2). However, the diameter size of the nanoparticles was different depending on the methodology used, in a range between 4 to 15 nm. The **CuNPs@CALB-1** showed the smallest nanoparticles (around 4 nm) whereas the **CuNPs@CALB-2** showed the largest (around 15 nm).

**Figure 3:**
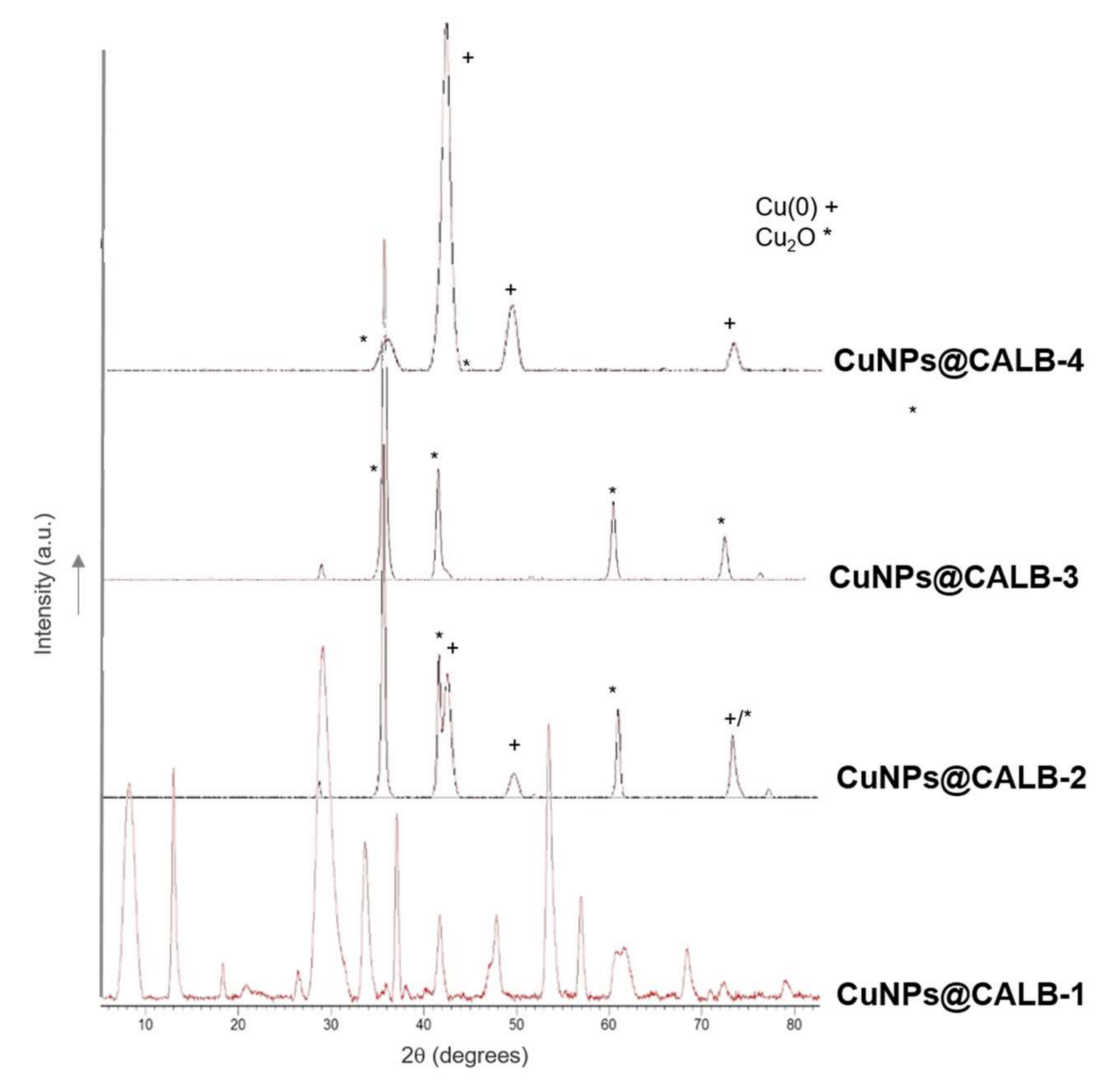
XRD pattern of the different CuNPs@CALB nanobiohybrids.

The XRD analysis was also performed in order to determine the copper species in each nanobiohybrid (Figure 3). The XRD pattern of **CuNPs@CALB-1** corresponded to the Cu_3_(PO_4_)_2_ species, which is well indexed (JCPDS card no. 22-0548). In the case of **CuNPs@CALB-2**, the XRD analysis revealed the presence of two copper species, mainly Cu^+^ in the form of Cu_2_O (matched well with JCPDS card no. 05-0667) and Cu (0) (matched well with JCPDS card no. 04-0836) in around 40%. However, **CuNPs@CALB-3** showed almost a unique copper species, Cu^+^ in the form of Cu_2_O. The difference in the synthetic protocol between these two latter nanobiohybrids, corresponding to the increase of the amount of protein in **CuNPs@CALB-3,** could control the final Cu species formation.

XRD of **CuNPs@CALB-4** showed the composition of mainly Cu (0) species, although around 20% of Cu_2_O is also detected in the sample.

The determination of cu amount on the solid in each case was determined by ICP-OES analyses. The percentages of copper in the different nanobiohybrids were 32%, 84%, 60%, and 93% for **CuNPs@CALB-1**, **CuNPs@CALB-2**, **CuNPs@CALB-3,** and **CuNPs@CALB-4** respectively.

### 3.2. Bisphenol A (BPA) Degradation by Cu Nanobiohybrids

An environmentally benign strategy in the degradation of BPA is the application of the Fenton method, by using an oxidative process in aqueous media and room temperature with hydrogen peroxide as a green oxidant.

**Figure 4:**
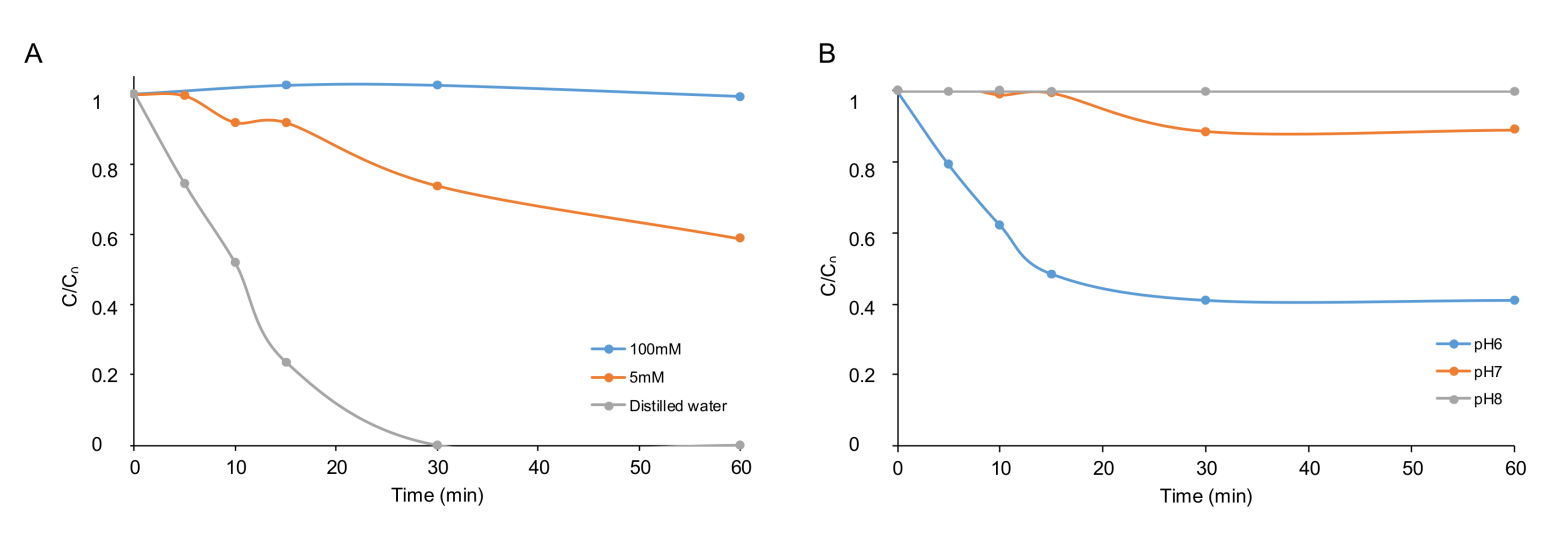
Hydrogen peroxide degradation profile of the **CuNPs@CALB-3** biohybrid at different experimental conditions in aqueous media. (**A**) Catalase activity at pH 8 in the presence of different ionic strength. (**B**) Catalase activity in 100 mM phosphate buffer at different pHs. Experimental conditions: [H_2_O_2_]: 50 mM, [catalyst]: 1.5 mg mL^−1^.

Recently, we found interesting results in the degradation of BPA by using an iron catalyst and H_2_O_2_ at acidic conditions (pH 4) [22]. Thus, the first trial tested these Cu nanobiohybrids at these conditions, however, these conditions were not appropriate for these catalysts (data not shown). The main reason for this instability was the vigorous formation of oxygen bubbles from the decomposition of the hydrogen peroxide. Thus, it was necessary to evaluate first the conditions for finding where no or highly reduced catalase activity of the Cu nanobiohybrids was observed. The H_2_O_2_ degradation in water was evaluated at three different pHs and directly in distilled water with 5 mM or 100 mM buffer solution (Figure 4, Figures S1–S3).

Figure 4 shows the results in H_2_O_2_ degradation of **CuNPs@CALB-3**. Different conditions of pHs were initially studied from pH 5 to 9 in distilled water, obtaining the best low H_2_O_2_ degradation results between 6–8 (data not shown). Thus, the effect of the ionic strength of the solution was evaluated in the catalase activity of the Cu nanobiohybrid (Figure 4A). It was clearly shown that the presence of buffer strongly reduced the catalase activity of this biohybrid, being completely eliminated using 100 mM buffer at pH 8.

Then, the combination of this buffer at different pHs showed how at pH 6, still around 60% catalase activity is maintained, while at pH 7, it almost disappeared, the best conditions being at pH 8.

Similar results were observed in the case of the other catalysts (Figures S1–S3).

Therefore, the degradation of BPA catalyzed by the different Cu biohybrids was evaluated at pH 7–8 in 100 mM aqueous buffer solution at room temperature using 100 mM H_2_O_2_ (Table 1).

The degradation rate (C_BPA_/C_0_) achieved at an initial point of 10 min determined great differences between the different catalysts (Table 1). Considering the amount of Cu in each case, the **CuNPs@CALB-3** was the faster catalyst with the highest TOF value at pH 8, degrading almost 90% of 45 ppm of BPA (Table 1, entry 6), which is 1.5-fold higher than using **CuNPs@CALB-2** (Table 1, entry 4) and 2.5- and almost 3-fold higher than **CuNPs@CALB-4** and **CuNPs@CALB-1** respectively. Decreasing the pH from 8 to 7 also caused an effect on the degradation degree, in most cases producing a fall in the catalytic performance except in **CuNPs@CALB-1** where the biohybrid exhibited duplicate degradation rate at 10 min incubation (Table 1, entries 1–2). Thus, a complete degradation of BPA was possible in 60 min using **CuNPs@CALB-2** and **CuNPs@CALB-3** (Table 1).

**Figure 5:**
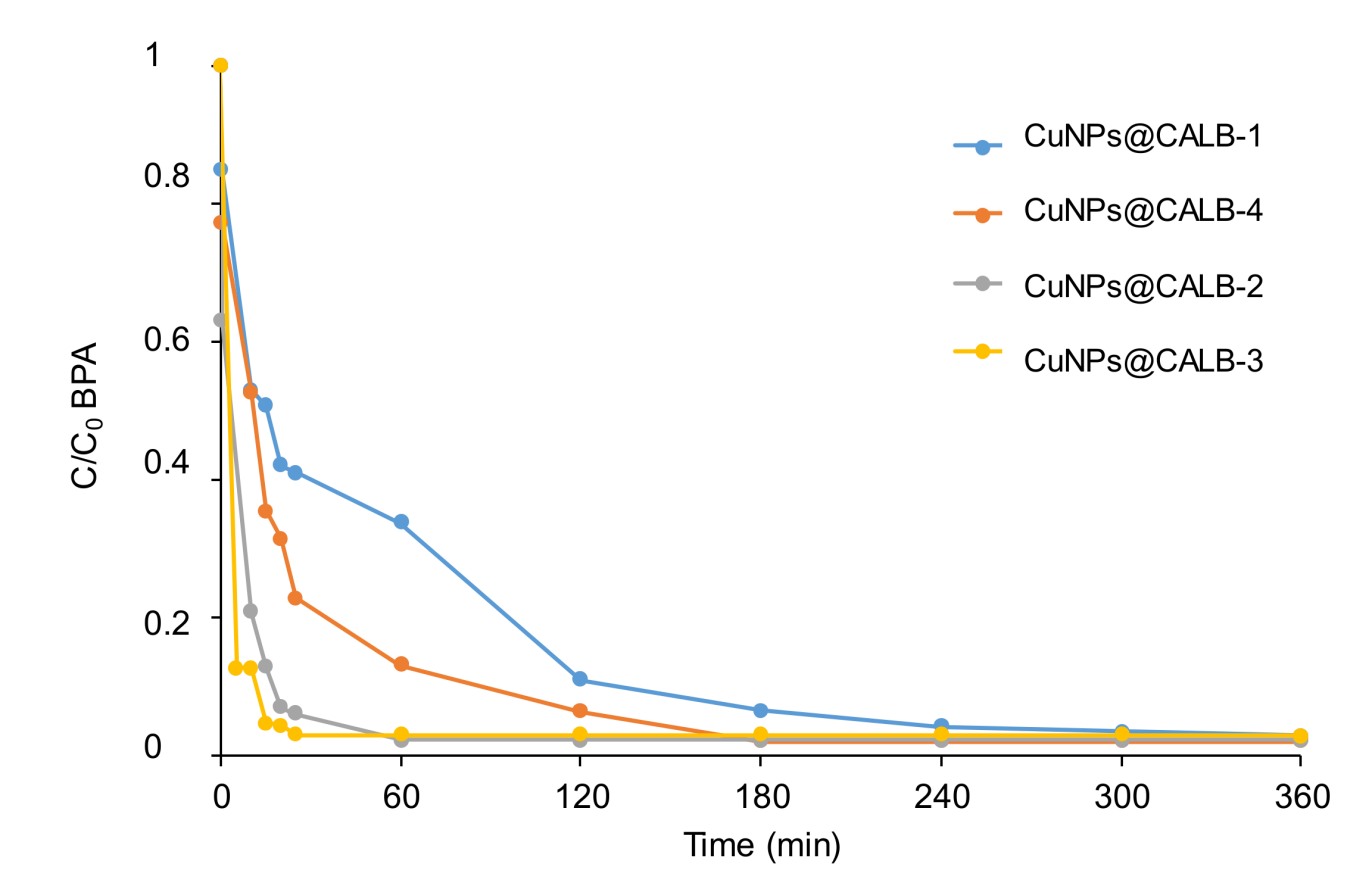
BPA degradation reaction profile catalyzed by the different CuNPs@CALB biohybrids. Experimental conditions: [BPA]: 45 mg L^−1^, [H_2_O_2_]: 100 mM, [Phosphate buffer]: 100 mM pH 8.

Furthermore, the complete BPA degradation profile was analyzed for all four nanobiohybrids (Figure 5).

The results in Figure 5 showed that very fast degradation is performed using **CuNPs@CALB-3**, where >95% BPA was degraded in 20 min. This represents, as far as we know, the fastest degradation process for eliminating BPA in aqueous media. [14,15,16] The **CuNPs@CALB-4** and **CuNPs@CALB-1** showed different profiles, although a similar time for final degradation for **CuNPs@CALB-4** was faster in the first 60 min (Figure 5).

These results demonstrate that Cu_2_O NPs species (**CuNPs@CALB-3**) are the best in catalytic performance on this reaction.

**Figure 6:**
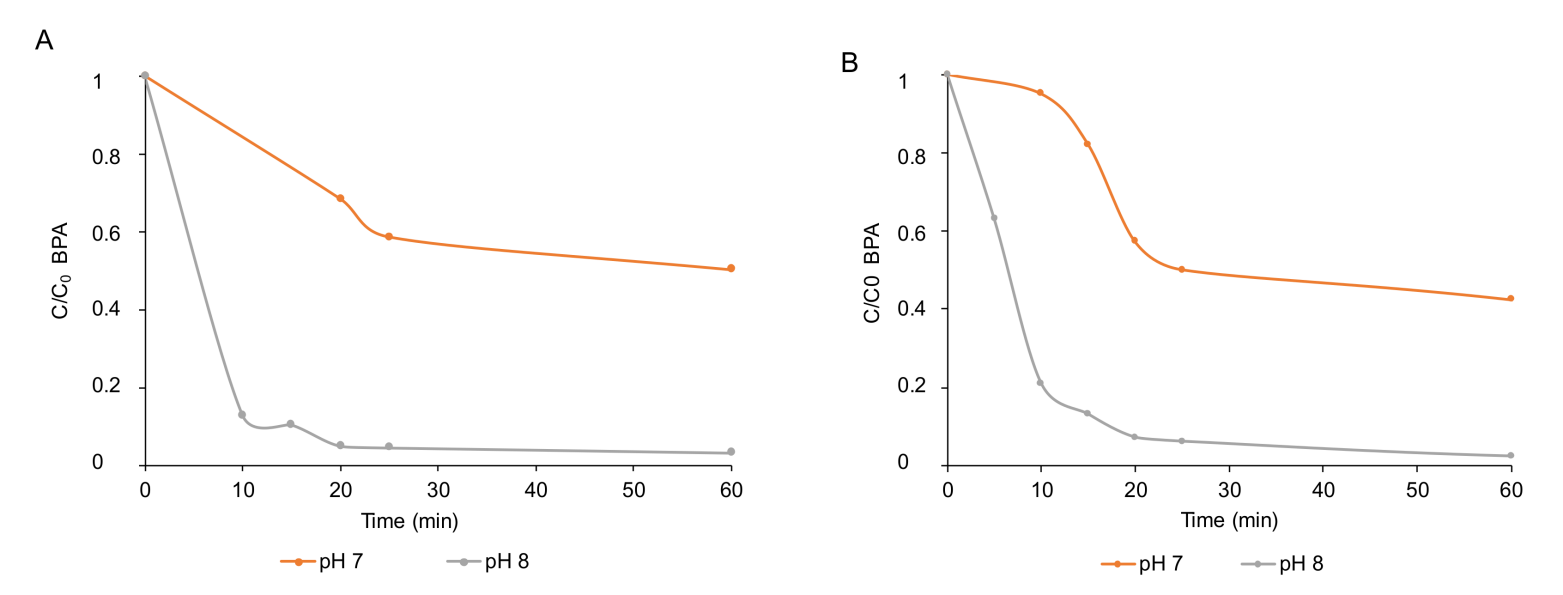
Effect of the pH on the BPA degradation. (**A**) **CuNPs@CALB-3**. (**B**) **CuNPs@CALB-2**. Experimental conditions: [BPA]: 45 mg L^−1^, [H_2_O_2_]: 100 mM, [Phosphate buffer]: 100 mM.

The reaction was also tested with these two excellent catalysts (**CuNPs@CALB-2** and **CuNPs@CALB-3**) at neutral pH in comparison with the previous pH conditions (Figure 6). The results demonstrated a clear effect on the catalytic degradation of BPA for both catalysts at neutral pH, importantly decreasing their efficiency slightly more for **CuNPs@CALB-3** (Figure 6A) with only 30% degradation after 20 min incubation at pH 7, whereas **CuNPs@CALB-2** degraded around 42% at this time at pH 7, with only 50% degradation being possible after 60 min (Figure 6).

**Figure 7:**
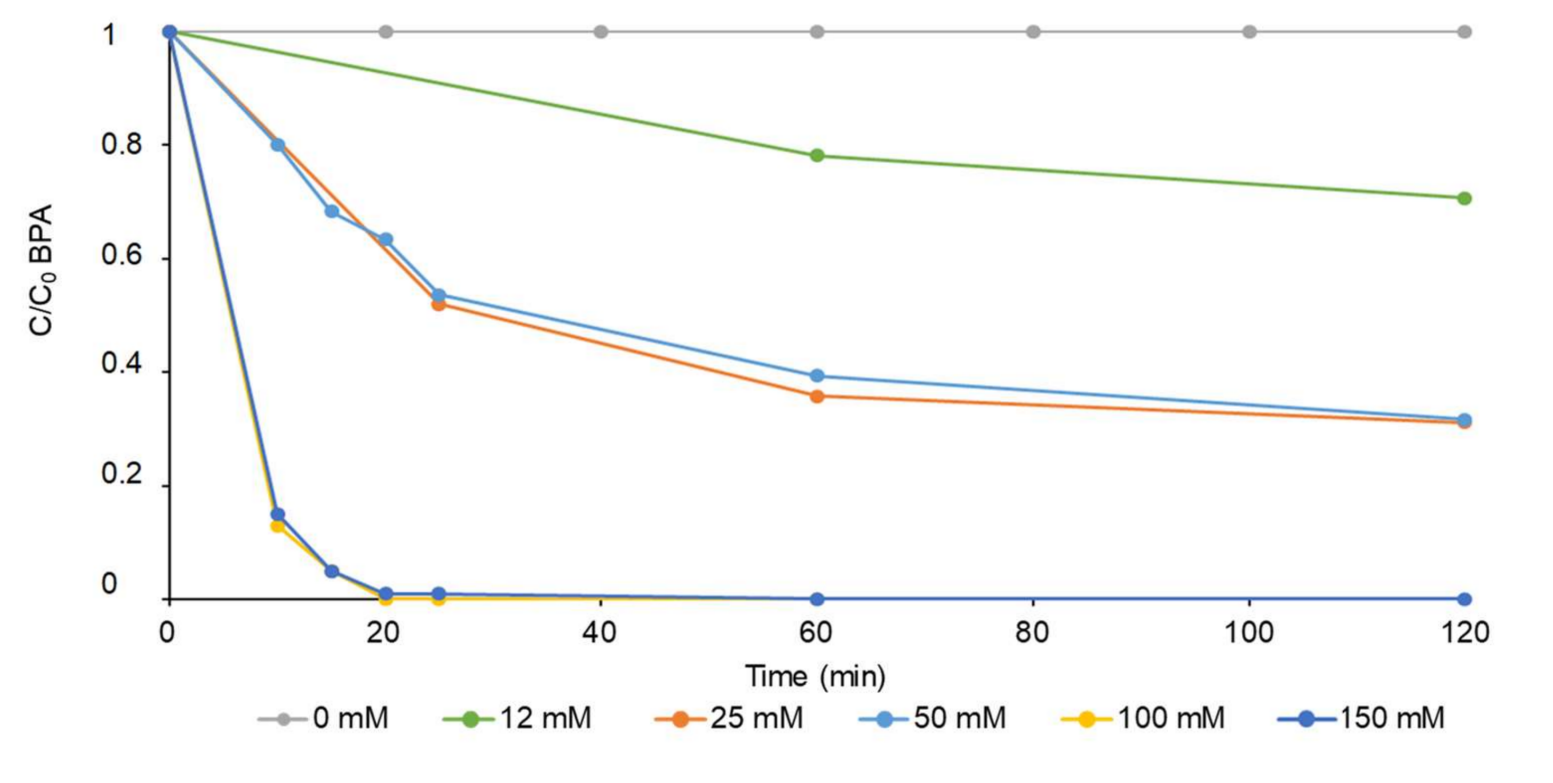
Effect of [H_2_O_2_] on the BPA degradation catalyzed by **CuNPs@CALB-3**. Experimental conditions: [BPA]: 45 mg L^-1^, [Phosphate buffer]: 100 mM pH 8.

Next, the amount of H_2_O_2_ as green oxidant added for the Fenton process was also evaluated. In this way, the BPA degradation reaction was performed at pH 8 using **CuNPs@CALB-3** as catalysts, and different concentrations of H_2_O_2_ from 0 to 150 mM were tested (Figure 7).

The degradation was very slow without H_2_O_2_, although the result was similar using 12 mM with around 20% degraded BPA in 120 min (Figure 7). The increase in the amount of the oxidant improved the degradation of **CuNPs@CALB-3**, although still using 25 or 50 mM of H_2_O_2_ less than 40% degraded BPA was obtained after 20 min reaction, around 60% degradation after 60 min. These results demonstrated that is necessary to have 100 mM of the oxidant for fast and complete degradation. Also, the addition of an extra amount of oxidant (150 mM) did not improve the results, which can conclude that 100 mM H_2_O_2_ seems to be the optimal amount for performing the reaction.

**Figure 8:**
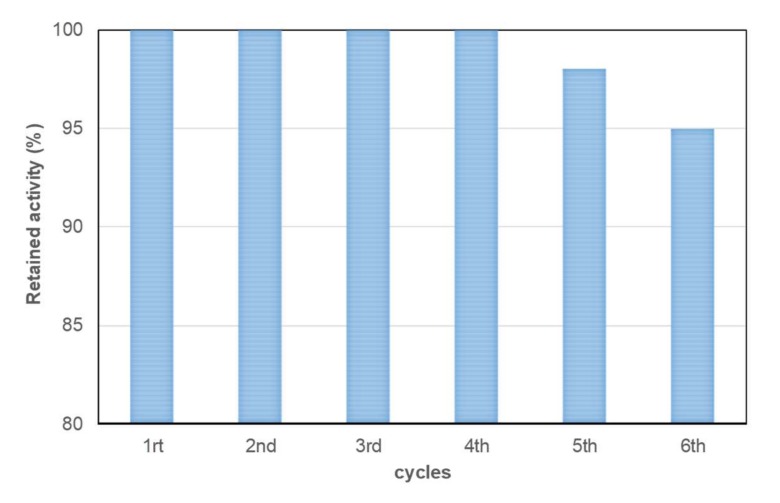
Recycling of **CuNPs@CALB-3** in oxidative degradation of BPA. Experimental conditions: [BPA]: 45 mg L^−1^, [H_2_O_2_]: 100 mM, [Phosphate buffer]: 100 mM pH 8.

Once optimal conditions were obtained, a recycling experiment was performed using the best Cu nanobiohybrid, **CuNPs@CALB-3** (Figure 8). The catalyst exhibited an excellent stability, maintaining 95% of the catalytic efficiency after five cycles of use.

Considering the very fast degradation process by this catalyst and in order to elucidate a possible mechanism of degradation of BPA, the reaction was performed reducing the ratio mg catalyst/mL reaction volume from 1.5 (the previous result) to 0.3. At these conditions, the **CuNPs@CALB-3** catalyst still worked well, degrading more than 95% BPA in 60 min (Figure S4).

Apart from this reduction in the amount of catalyst for the reaction, considering the limit in the international regulation of BPA concentration (0.05 ppm), an experiment reducing the amount of BPA from 45 up to 4.5 ppm was performed using **CuNPs@CALB-2** as the catalyst (Figure S5).

The reaction rate in BPA degradation did not increase significantly by twice reducing the BPA concentration; however, the catalyst was able to degrade >90% BPA in 25 min when 4.5 ppm of the substrate was used (Figure S5).

**Figure 9:**
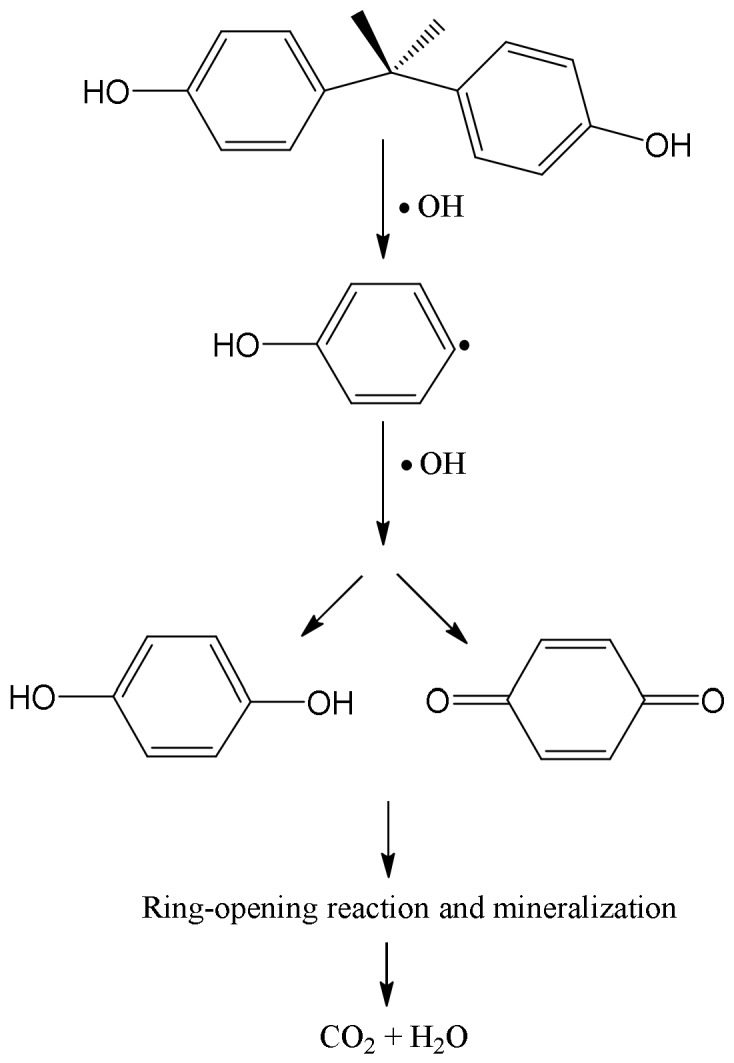
Proposed Mechanism of oxidative BPA degradation catalyzed by CuNPs@CALB biohybrids.

Finally, in order to explain a possible mechanism of BPA degradation by this Cu nanobiohybrids catalyst, we evaluated the reaction profile of the degradation of BPA by analyzing the reaction products formation by HPLC. In order to determine the possible degradation mechanism, the reaction was performed using 25 mM of hydrogen peroxide conditions where the reaction was much slower. At these conditions, benzoquinone was the main product observed for BPA degradation, which indicates that the first step was the BPA decomposition to an aromatic intermediate by direct oxidation. This mechanism is in concordance with a previous report published by Giu et al. [23] (Figure 9) and others [14,15] where probably the Cu catalyst generated a hydroxyl radical from the oxidation of hydrogen peroxide, which produces the phenol radical precursor of the benzoquinone. Repeating the reaction adding TEMPO on the solid phase clearly decreased the BPA degradation speed.

## 4. Conclusions

Four different Cu nanobiohybrids with different Cu species and nanoparticle sizes were used as catalysts in the degradation of BPA in water.

The effect on the final catalytic efficiency in the degradation process was determined depending on the copper species, being the biohybrid **CuNPs@CALB-3**, which contained Cu_2_O nanoparticles of around 10 nm size, the highly efficient one of all tested. It was able to eliminate more than 95% of BPA (45 ppm) in 20 min in the presence of hydrogen peroxide at pH 8 using 1.5 g/L of a catalyst. The catalyst showed excellent stability and recyclability at these conditions.

These excellent results open the possibility to extend the application of these Cu nanocatalysts in environmental technology in the degradation of other toxic organic pollutants.

## Figures and Tables

**Table 1:** BPA degradation catalyzed by different CuNPs@CALB biohybrids ^a^

Entry	Nanocatalyst	pH	BPA Degradation at 10 min (%)	TOF ^b^ (h^−1^)	t_conv_ >99% ^c^ (min)
1	**CuNPs@CALB-1**	7	20.63	240	1140
2	**CuNPs@CALB-1**	8	11.30	126	1020
3	**CuNPs@CALB-2**	7	18.00	60	>1500
4	**CuNPs@CALB-2**	8	79.10	246	60
5	**CuNPs@CALB-3**	7	31.53	66	>1500
6	**CuNPs@CALB-3**	8	87.13	366	60
7	**CuNPs@CALB-4**	7	39.96	120	1020
8	**CuNPs@CALB-4**	8	47.33	144	1020

^a^ Experimental conditions: [BPA]: 45 mg L^−1^, [H_2_O_2_]: 100 mM, [Phosphate buffer]: 100 mM pH 8. ^b^ TOF value × 10^−3^. ^c^ Time to achieve a full BPA degradation.

## Supplementary Materials

The following are available online at https://www.mdpi.com/2079-4991/10/1/7/s1, Figure S1, Figure S2 and Figure S3: Hydrogen peroxide degradation profile of the CuNPs@CALB-1, CuNPs@CALB-2 and CuNPs@CALB-4 biohybrids respectively, Figure S4: Degradation of BPA catalyzed by CuNPs@CALB-3 changing the relation catalyst amount/reaction volume, Figure S5: Degradation of BPA catalyzed by CuNPs@CALB-2 at different BPA concentrations.
