# Supplementary material for: Fast Degradation of Bisphenol A in Water by Nanostructured CuNPs@CALB Biohybrid Catalysts
Source: Nanomaterials (Basel). 2019 Dec 18;10(1):7. doi: 10.3390/nano10010007 (PMC7023019; doi:10.3390/nano10010007)
Supplement: Supplementary file 1 [file nanomaterials-10-00007-s001.pdf]

Supplementary Material

Fast Degradation of Bisphenol A in Water by Nanostructured CuNPs@CALB Biohybrid Catalysts

Noelia Losada-Garcia, Alba Rodriguez-Otero and Jose M. Palomo *

Department of Biocatalysis, Institute of Catalysis (CSIC), Marie Curie 2, Cantoblanco, Campus UAM, 28049 Madrid, Spain; n.losada@csic.es (N.L.-G.); alba.rodriguez.96@gmail.com (A.R.-O.)

* Correspondence: josempalomo@icp.csic.es; Tel.: +34-9158-5476-8

A

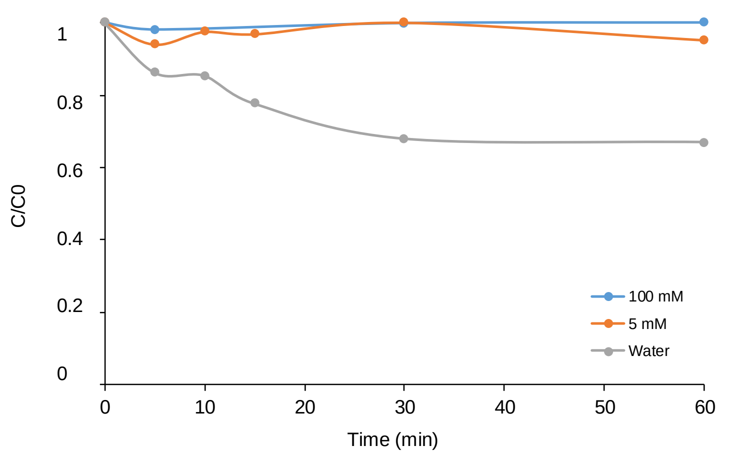

B

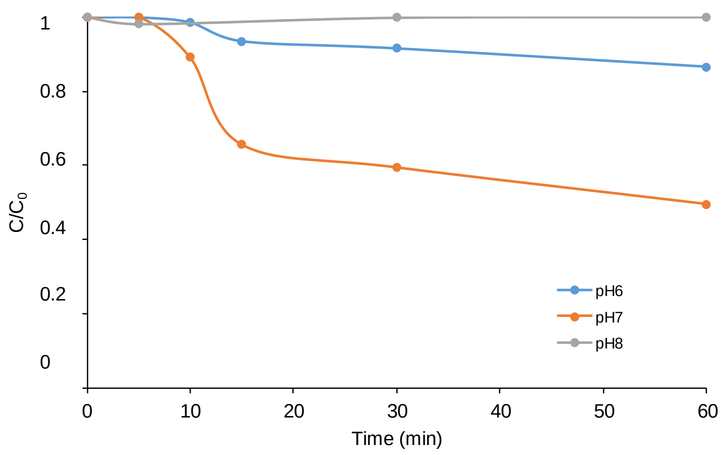

Figure S1. Hydrogen peroxide degradation profile of the **CuNPs@CALB-1** biohybrid at different experimental conditions in aqueous media. A) Catalase activity at pH 8 in the presence of different ionic strength. B) Catalase activity in 100 mM phosphate buffer at different pHs. Experimental conditions: [H_2O_2]: 50 mM, [catalyst]:1.5 mg. mL^{-1}.

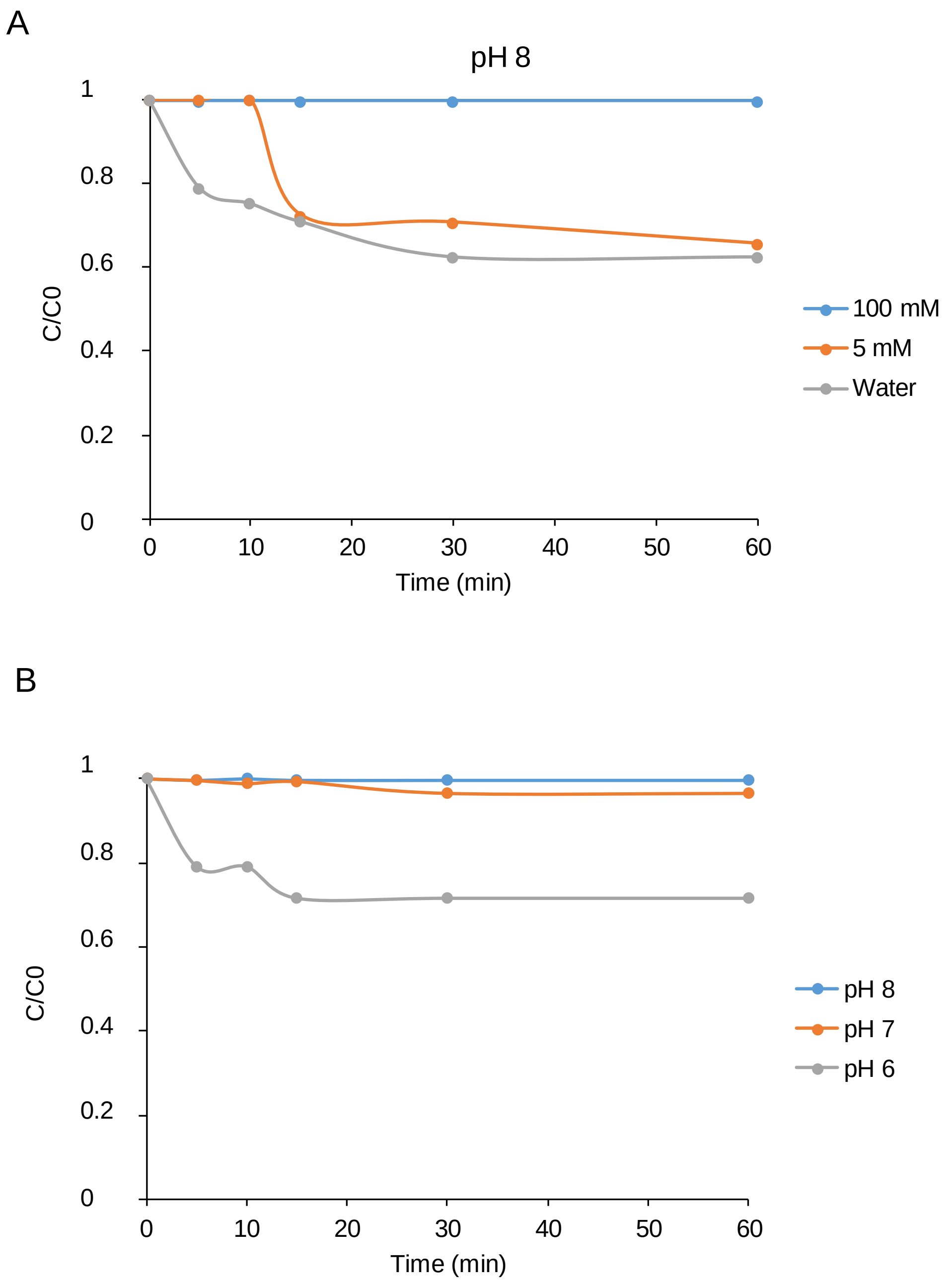

Figure S2. Hydrogen peroxide degradation profile of the **CuNPs@CALB-2** biohybrid at different experimental conditions in aqueous media. A) Catalase activity at pH 8 in the presence of different ionic strength. B) Catalase activity in 100 mM phosphate buffer at different pHs. Experimental conditions: [H_2O_2]: 50 mM, [catalyst]:1.5 mg. mL^{-1}.

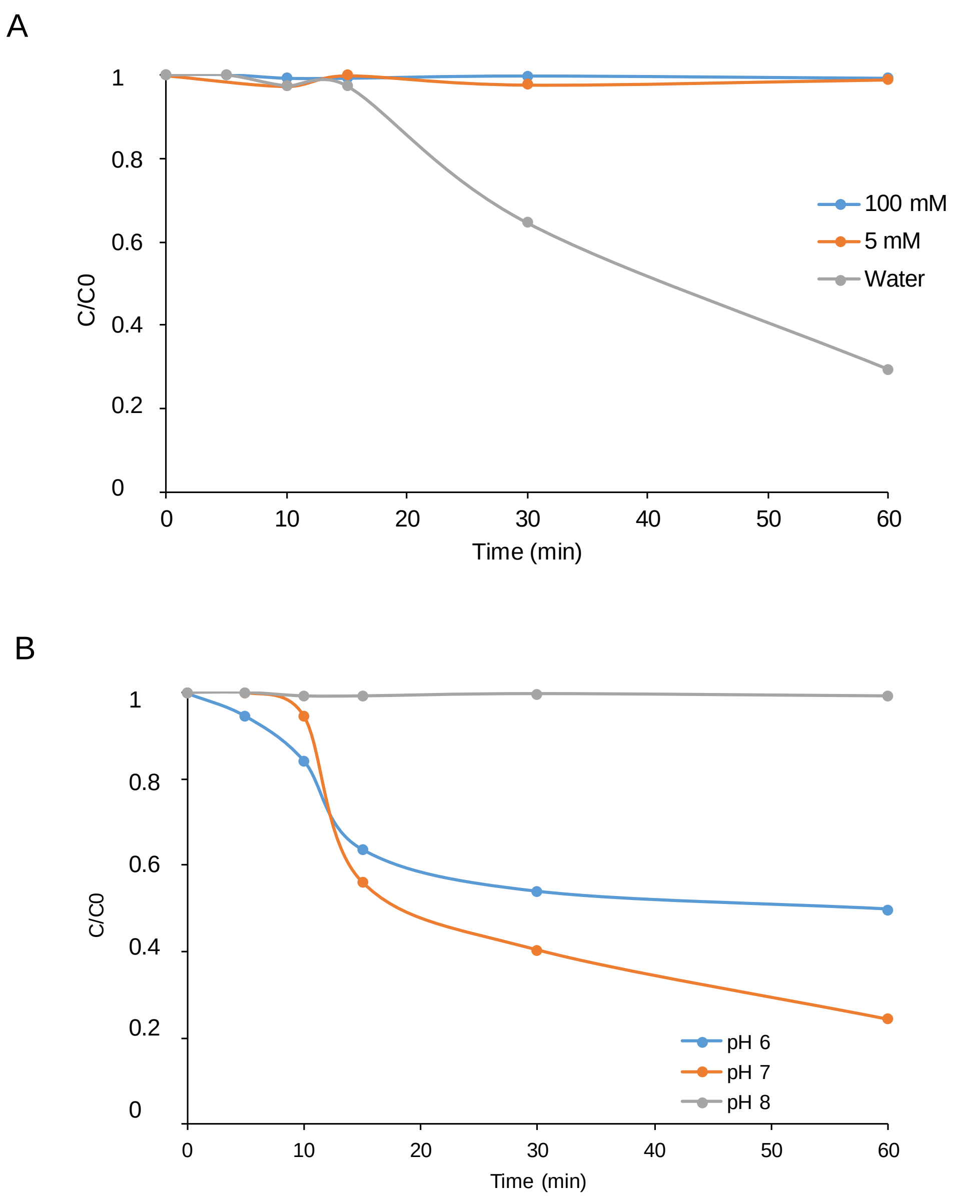

Figure S3. Hydrogen peroxide degradation profile of the **CuNPs@CALB-4** biohybrid at different experimental conditions in aqueous media. A) Catalase activity at pH 8 in the presence of different ionic strength. B) Catalase activity in 100 mM phosphate buffer at different pHs. Experimental conditions: [H_2O_2]: 50 mM, [catalyst]:1.5 mg. mL^{-1}.

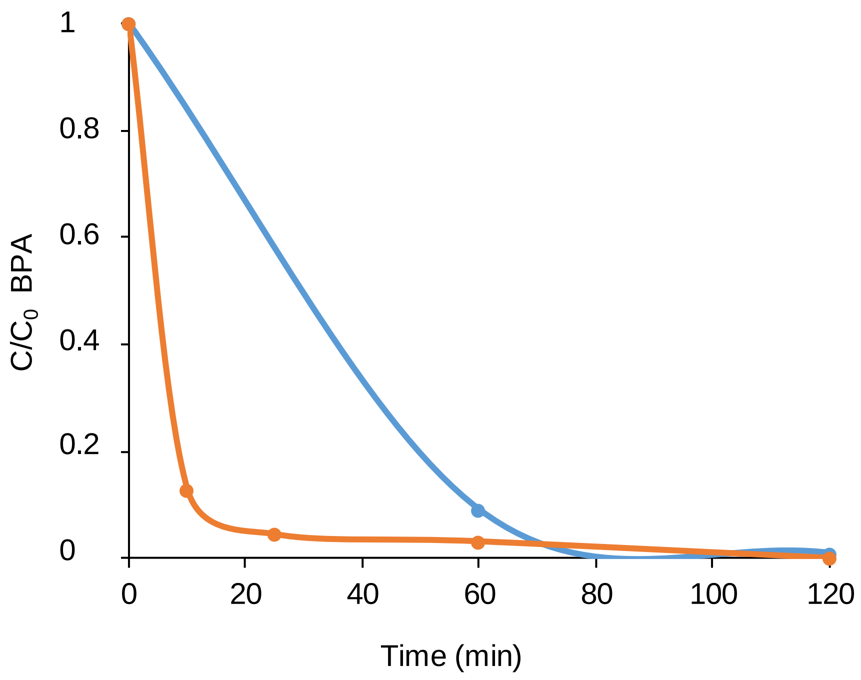

Figure S4. Degradation of BPA catalyzed by **CuNPs@CALB-3** changing the relation catalyst amount/ reaction volume. Experimental conditions: [BPA]: 45 mg. L^{-1}, [H_2O_2]: 100 mM, [Phosphate buffer]:100 mM pH 8. [catalyst]:1.5 mg. mL^{-1} (orange) or 0.3 mg. mL^{-1} (blue).

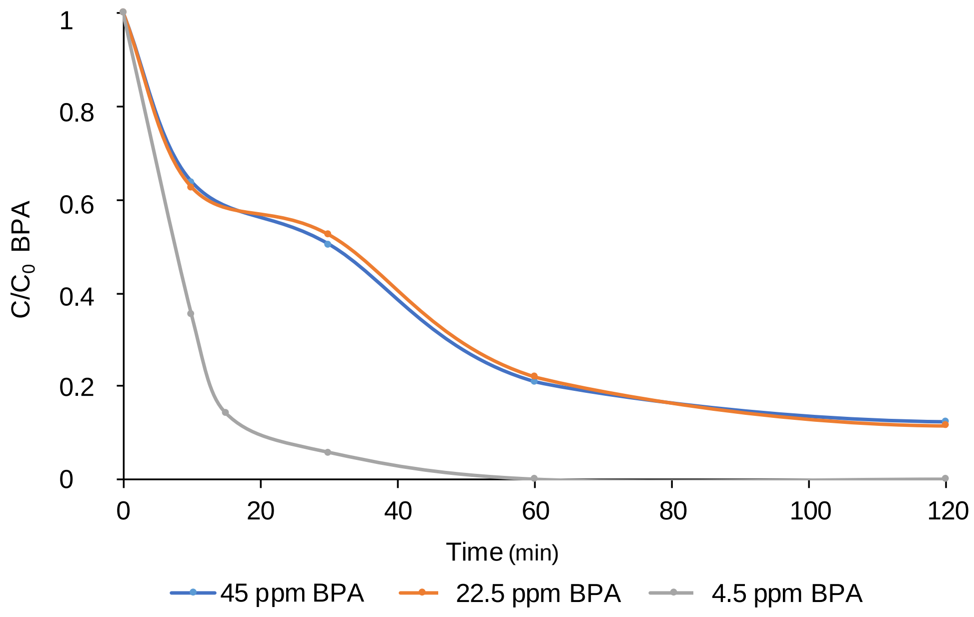

Figure S5. Degradation of BPA catalyzed by **CuNPs@CALB-2** at different BPA concentrations. Experimental conditions: [H_2O_2]: 100 mM, [Phosphate buffer]:100 mM pH 8. [catalyst]:1.5 mg. mL^{-1}.
